# Magnon-tuning non-volatile magnetic dynamics in a CoZr/PMN-PT structure

**DOI:** 10.1038/s41598-020-71409-9

**Published:** 2020-09-01

**Authors:** Cai Zhou, Ming-fang Zhang, Fu-fu Liu, Ying Jin, Chang-jun Jiang, Min Hu, Cun-fang Feng, Feng-long Wang, Ming-yao Xu, Sheng-xiang Wang

**Affiliations:** 1 Hubei Province Engineering Research Center for Intelligent Micro-Nano Medical Equipment and Key Technologies, Wuhan Textile University, Wuhan, 430200 People’s Republic of China; 2 Key Laboratory for Magnetism and Magnetic Materials, Ministry of Education, Lanzhou University, Lanzhou, 730000 People’s Republic of China; 3 Hubei Engineering and Technology Research Center for Functional Fiber Fabrication and Testing, Wuhan Textile University, Wuhan, 430200 People’s Republic of China; 4 Department of Applied Physics, Chang’an University, Xi’an, 710064 People’s Republic of China

**Keywords:** Materials science, Physics

## Abstract

Magnon-tuning non-volatile magnetic dynamics is investigated in a CoZr/PMN-PT structure by measuring ferromagnetic resonance at room temperature. The electric-field control of ferromagnetic resonance shows *loop-*like behavior, which indicates non-volatile electric-field control of the magnetism. Further, fitting the curves of in-plane rotating angle versus ferromagnetic resonance field under different electric fields shows that the effective magnetic field changes in *loop-*like manner with the electric field. The resulting change in non-volatile saturation magnetization with electric field is consistent with that of a polarization electric field curve. A 1.04% change of saturation magnetization is obtained, which can be attributed to a magnon-driven magnetoelectric coupling at the CoZr/PMN-PT interface. This magnon-driven magnetoelectric coupling and its dynamic magnetic properties are significant for developing future magnetoelectric devices.

## Introduction

Controlling the electric field in magnetism has the potential to boost spintronics in ferromagnetic/ferroelectric (FM/FE) multiferroic heterostructures^1,2^. In an FM/FE system with interfacial coupling between magnetization and electric polarization, the magnetic dynamics can be mediated by the electric field, in which case the electric field may manipulate the charge carrier density and/or affect the magnetic moment and magnetic anisotropy^3–8^. Further, interfacial charge accumulation or depletion can lead to a non-volatile magnetic response to the electric field^9^. The conventional charge screening length is on the order of an angstrom^10–13^. However, interfacial charge of the FM layer is spin-polarized when the electric field is applied to an itinerant FM layer. A local nonuniform spiral spin density builds up at the interface because the spin-polarization screening charge is surface confined, leading to an initial uniform magnetization away from the interface. This produces a strong and linear magnetoelectric (ME) coupling that is noticeable near the itinerant FM interface. Moreover, the range of spiral spin density can reach the order of nanometers, which has a clear advantage in allowing non-volatile device for information storage. This can be viewed as magnonic accumulation that is stabilized by the charge rearrangement between the coupled FE (with an FE polarization **P**) and FM (with an FM magnetization **M**)^14–20^. Thus, it is predicted that the magnon-driven interfacial ME coupling-induced changes in the magnetic anisotropy will lead to elastic magnetic dynamics in the FM layer. Accordingly, in this work, a polycrystalline CoZr (Co_96_Zr_4_) layer is sputtered on a single-crystalline PMN-PT (Pb(Mg_1/3_Nb_2/3_)_0.7_Ti_0.3_O_3_) substrate through magnetron sputtering. The magnon control of magnetic dynamics in a CoZr/PMN-PT structure is investigated by measuring ferromagnetic resonance (FMR) at room temperature.

## Results

**Figure 1:**
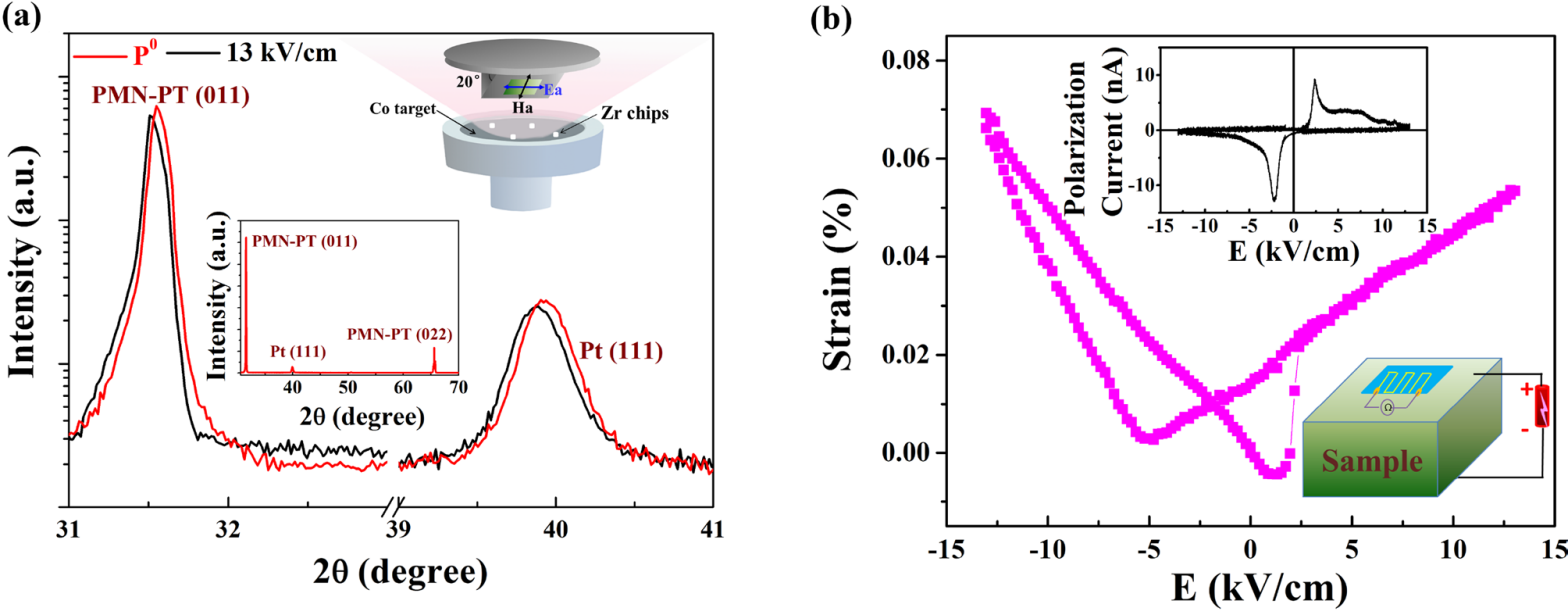
(**a**) XRD 2θ scanning patterns at P^0^ state and under the electric field 13.0 kV/cm. The inset shows the schematic of the sputtering arrangement. (**b**) S–E curve. The top inset shows polarization–electric-field curve and thus the electric field tuning of the in-plane polarization current. The bottom inset is a sketch of the strain gauge measurement.

Figure 1a shows the out-of-plane θ–2θ XRD scan of the 20-nm CoZr/PMN-PT structure at P^0^ state. The term P^0^ represents the initial state, in which the crystal is unpolarized. The prominent PMN-PT (011) and (022) peaks are recorded at 31.5° and 65.5°, respectively, which shows the PMN-PT substrate with (011)-orientation^21–23^. The (111) peak of the Pt layer is also present at 39.9°, while there is no CoZr peak in the XRD scans. The phase of the CoZr layer can be considered polycrystalline according to previous work^24^. Remarkably, (011) peak of PMN-PT substrate shift toward lower angles under the electric field 13.0 kV/cm, which reveals an expansion along out-of-plane direction. Moreover, a (111) peak of Pt layer also shift toward lower angles, which indicates the PMN-PT substrate produced an out-of-plane tensile strain in the Pt layer associated with applying electric field. The change of out-of-plane lattice parameters of PMN-PT and Pt with application of electric field are estimated as a similar shift of 0.14%^7,22^. The strain was measured with a platform we designed in which the CoZr/PMN-PT structure was stuck on a strain gauge (KFG-A-120-d17, KYOWA) with special glue (CC-33A) as shown in the bottom inset of Fig. 1b. The positive electric field direction was defined as being from top to bottom, whereas negative. The electric field applied on the PMN-PT (011) single-crystalline can produce biaxial in-plane strain. When applying electric field along the [011] direction of PMN-PT, the direction of in-plane compressive stress is along [100] direction of PMN-PT, while the direction of tensile stress is along [01− 1] direction of PMN-PT^25–27^. A *butterfly*-like strain–electric-field (S–E) curve was obtained as shown in Fig. 1b, which is measured along the [01− 1] direction of PMN-PT. The corresponding polarization current curve was shown in the top inset of Fig. 1b, which reveals that the coercive electric field is approximately 2.2 kV/cm. This indicates that the polarization charge can be mediated by the electric field, which leads to the interfacial ME coupling in the CoZr/PMN-PT structure.

**Figure 2:**
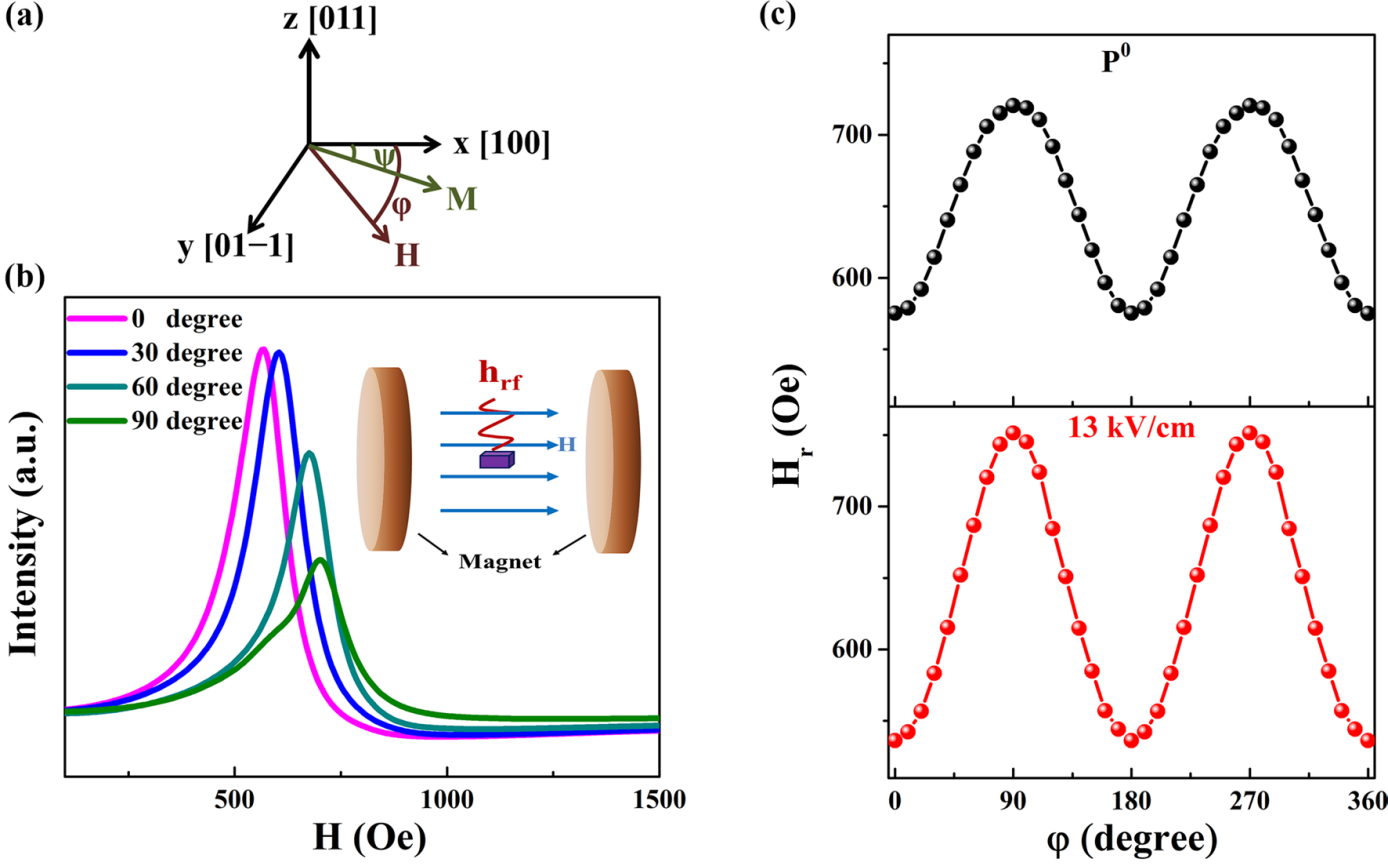
Dynamic magnetic properties at P^0^ state and under a electric field of 13.0 kV/cm. FMR integral spectra (**a**) Schematic of field direction during FMR measurement. (**b**) FMR integral spectra with H parallel to the films in-plane at different oblique sputtering angles at P^0^ state. The inset shows the configuration for FMR spectroscopy. (**c**) The H_r_–φ curves at P^0^ state and under an electric field of 13.0 kV/cm.

The dynamic magnetic property of the CoZr/PMN-PT structure was investigated through FMR measurement at room temperature. Figure 2a shows schematic of the field direction during FMR measurement. Therein, φ is the angle between the direction of the applied magnetic field and the [100] direction of PMN-PT, and ψ is the angle between the direction of magnetization and the [100] direction of PMN-PT. The configuration for FMR spectroscopy is shown in the inset of Fig. 2b. The sample had a 1 mm × 1 mm square shape. Two Cu wires were connected to the top and bottom Pt electrodes of the CoZr/PMN-PT structure, then fixed them by insulating tape. The dc electric field provided by a Keithley 2,410 dc power supply was applied on the two Cu wires. FMR absorption spectra were measured using a lock-in technique based on sweeping a static external magnetic field superimposed over the AC magnetic field. The in-plane rotating-angle FMR integral spectra of the CoZr/PMN-PT structure were obtained for the P^0^ state at 0°, 30°, 60° and 90° as shown in Fig. 2b. Because the magnetization was probed using a special phase correlation under the microwave excitation, the spectra in fact correspond to a mixture of imaginary and real parts. Therefore, the actual function of the FMR integral curve is given as^4,16,17^:

1 $$ \zeta (H) = A\frac{{\Delta H\cos \delta + (H - H_{r} )\sin \delta }}{{\Delta H^{2} + (H - H_{r} )^{2} }} $$

where A is the integral coefficient, ΔH is the half-width at half-maximum, H_r_ is the magnetic resonance field, δ is the phase that mixes the real and imaginary parts of the dynamic susceptibility, and H is the external magnetic field. The FMR integral curves can be fitted according to the Eq. (1) to obtain related parameters, especially H_r_. The H_r_–φ curve was obtained for the P^0^ state as shown in the top image of Fig. 2c, which demonstrates that the CoZr layer has an in-plane uniaxial anisotropy originating from the oblique magnetron sputtering. At 0° and 180°, H_r_ is at its minimum, implying the direction of the easy magnetization axis. Meanwhile, the maxima of H_r_ at 90° and 270° represent the direction of the hard magnetization axis. Similarly, the H_r_ − φ curve under an electric field of 13.0 kV/cm was obtained as shown in the bottom image of Fig. 2c. The directions of the easy and hard magnetization axes remain the same under the applied electric field. However, H_r_ changes with the electric field, which can be attributed to the interfacial ME coupling in the CoZr/PMN-PT structure.

**Figure 3:**
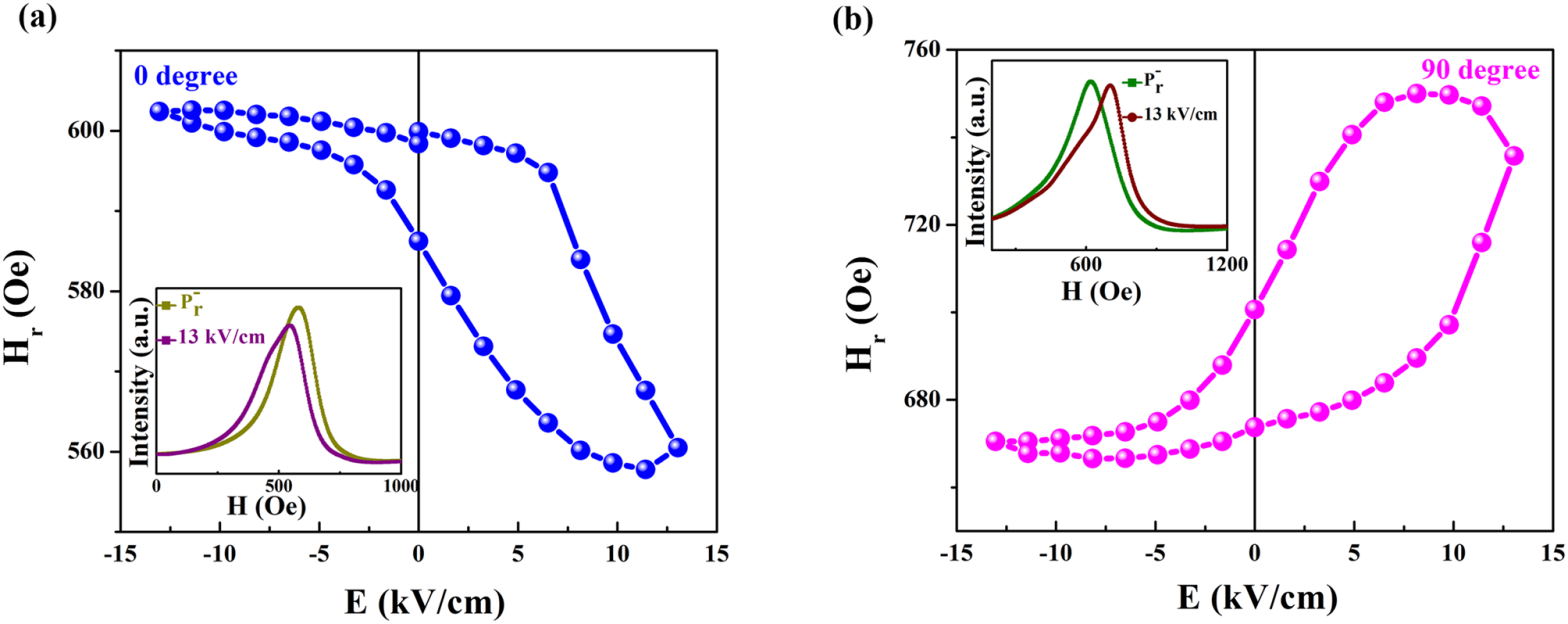
H_r_–E curves at 0° and 90°. H_r_–E curves at (**a**) 0° and (**b**) 90°. The inset shows the FMR integral spectra at P_r_^−^ state and under an electric field of 13.0 kV/cm.

To investigate the possible interfacial ME coupling, the FMR integral curves under different electric fields were measured at 0° and 90°, respectively. The resulting fitted H_r_–E curve is shown in Fig. 3. For the result at 0° shown in Fig. 3a, the inset shows the FMR integral spectra at P_r_^−^ state and under an electric field of 13.0 kV/cm. The states P_r_^+^ and P_r_^−^, are the two remnant polarization states after the applied electric fields of 13.0 and − 13.0 kV/cm are turned off, respectively. Under the positive electric field, H_r_ decreases sharply when the electric field is swept from the P_r_^−^ state to 13.0 kV/cm, and then increases linearly when the electric field changes from 13.0 kV/cm to the P_r_^+^ state. Under the negative electric field, the H_r_ dependence on the electric field from the P_r_^+^ state to − 13.0 kV/cm slightly increases, and then remains unchanged until the P_r_^−^ state. The *loop*-like H_r_–E curve obtained at 0° reveals the non-volatile electric field control of magnetic behavior. Meanwhile, the result at 90° is similar to that in Fig. 3b. There are generally two main ME coupling mechanisms, the piezostrain effect and charge effect, in FM/FE multiferroic heterostructures. The S–E curve for the CoZr/PMN-PT structure in Fig. 1b shows that a piezostrain effect can be transferred to the CoZr layer to achieve a *butterfly*-like magnetic response to the electric field. Compared with the FMR results in Fig. 3, the non-symmetric *loop*-like H_r_–E curves demonstrate non-volatile behavior, which is different from the *butterfly*-like S–E curve. This indicates that the piezostrain effect does not play a key role in the interfacial ME effect. The magnetic dynamic result of the coupling phenomena should be considered with care. However, the charge screening length for the conventional charge-mediated mechanism is only a few angstroms. In FeNi/PMN-PT structures and MgO-based magnetic tunnel junctions^10,11^, the charge-mediated ME effect is negligible in relatively thick FM layers (≥ 3 nm). Weisheit. et. al. have reported that the magnetocrystalline anisotropy of ordered FePd can be modified by an applied electric field. The strong influence of the surface on the FePd material can be observed only when the thickness of the FePd layer is less than 2 nm^12^. Yi et al. have investigated the charge-modulated ME coupling in a La_0.7_Sr_0.3_MnO_3_/BiFeO_3_ heterostructure through interface engineering, where the thickness of the La_0.7_Sr_0.3_MnO_3_ layer is only 13 unit cells^28^. The thickness of the CoZr layer in our CoZr/PMN-PT structure is 20 nm, and thus the conventional charge-mediated ME coupling can be ignored, considering that the diffusion length of magnon-driven interfacial ME coupling can reach several tens of nanometers. Moreover, this magnon-driven interfacial ME coupling has a strong correlation between the polarization **P** and magnetization **M** as shown in the top inset of Fig. 5, which leads to a *loop*-like magnetic response to the electric field such as the H_r_–E curve as shown in Fig. 3. Therefore, the magnon-driven ME coupling has a predominant effect in the CoZr/PMN-PT structure.

**Figure 4:**
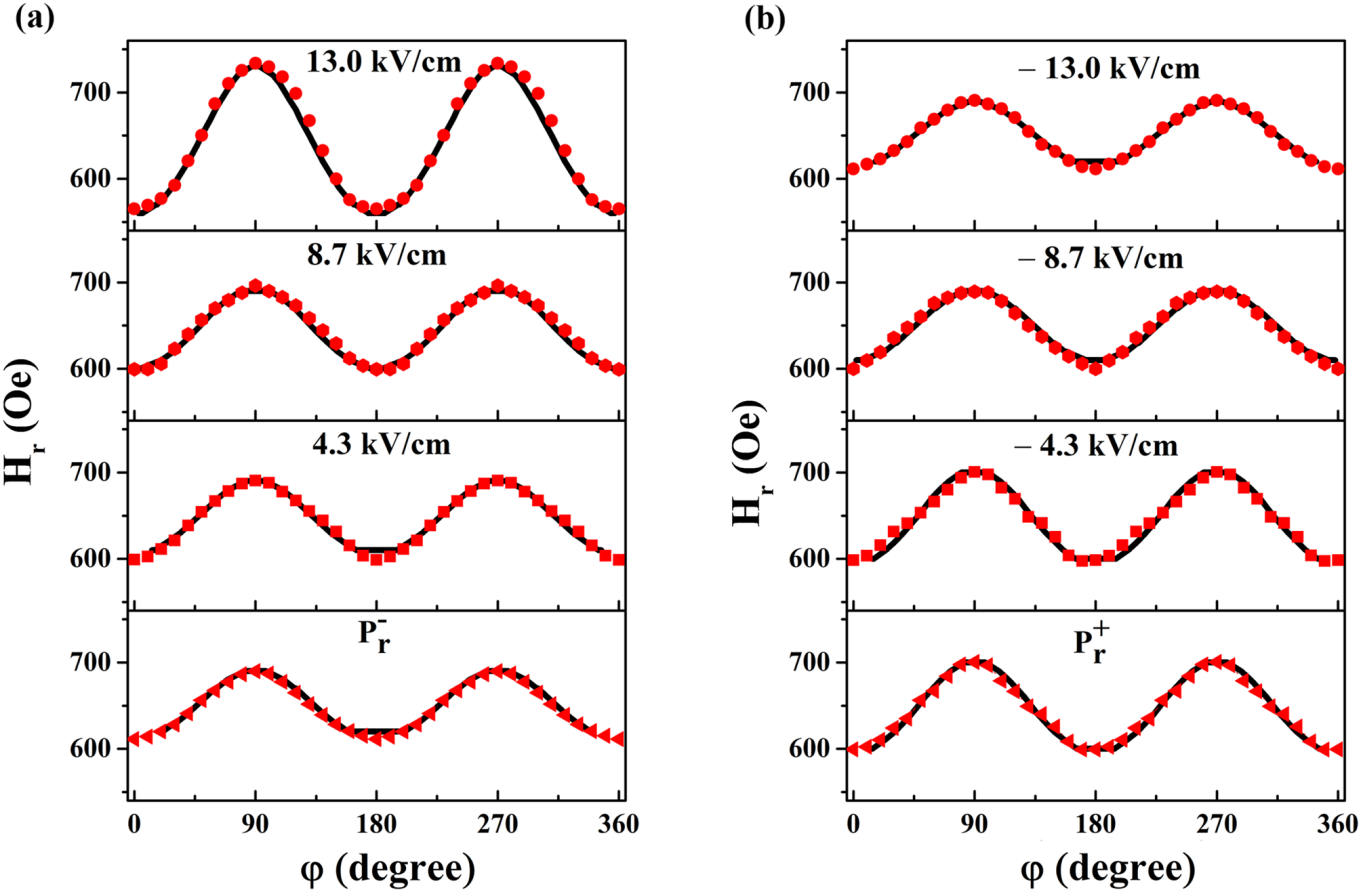
Experimental and fitted H_r_–φ curves. Experimental H_r_–φ curves (red dots) under (**a**) positive and (**b**) negative electric fields along with the fitted H_r_–φ curves (black lines).

To further verify the magnon-driven interfacial ME coupling in the CoZr/PMN-PT structure, we plot the experimental H_r_ dependence on φ under different electric fields in Fig. 4 (red dots), which can be fitted using the equation of the in-plane measurement configuration^29^:

2 $$ \left( {{\omega \mathord{\left/ {\vphantom {\omega \gamma }} \right. \kern-\nulldelimiterspace} \gamma }} \right)^{2} = \left\{ {H_{r} \cos (\psi - \varphi ) + 4\pi M_{s} + H_{eff} \cos^{2} \varphi } \right\} \cdot \left\{ {H_{r} \cos (\psi - \varphi ) + H_{eff} \cos 2\varphi } \right\} $$

**Figure 5:**
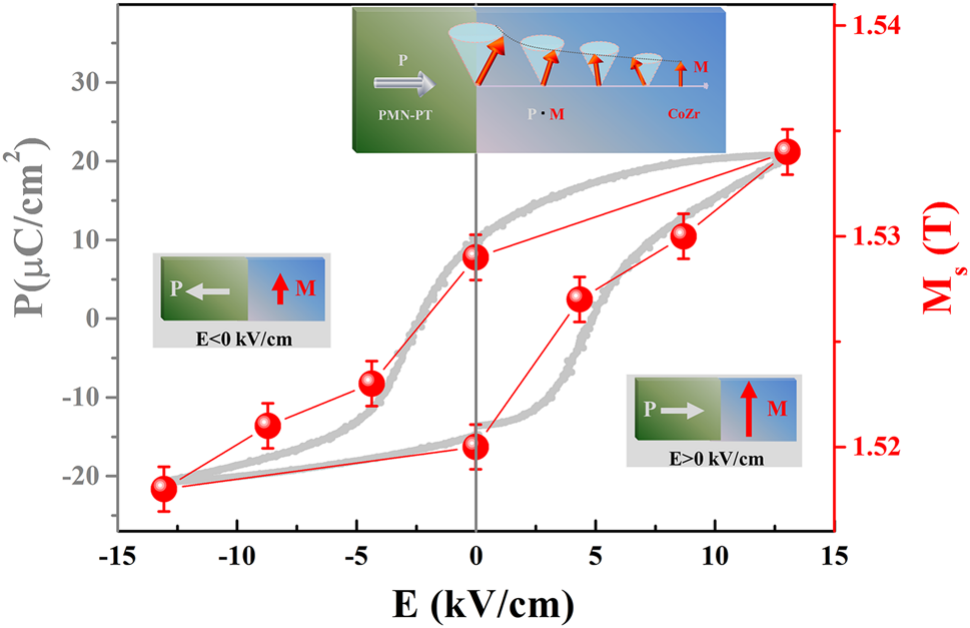
M_s_–E and P–E curves. The inset illustrates the P–M interaction.

**Figure 6:**
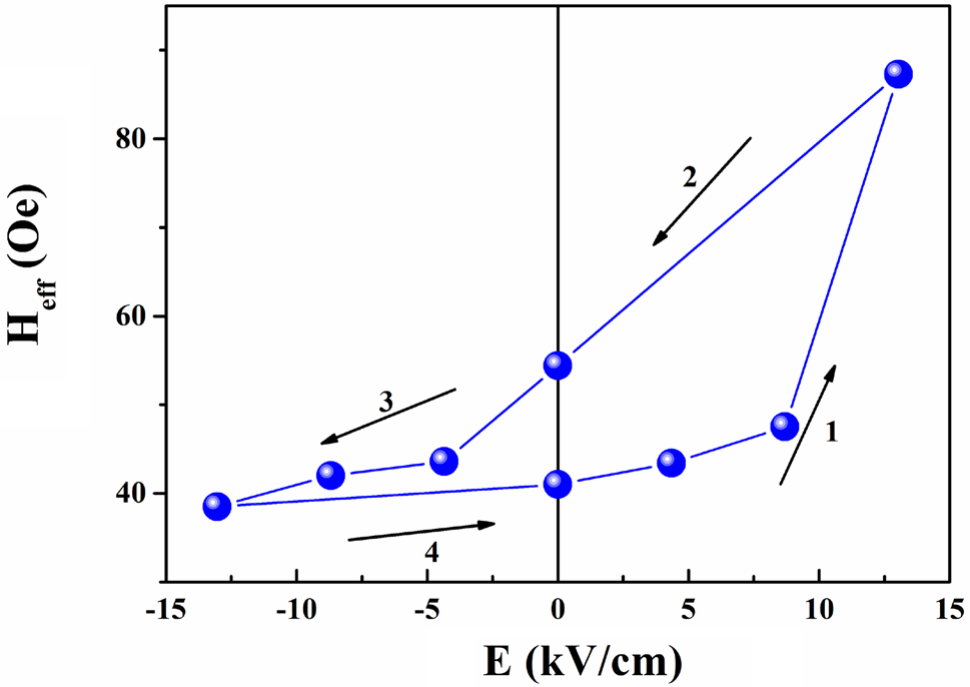
H_eff_–E curve.

We take into account magnetization equilibrium:

3 $$ H_{r} \sin (\psi - \varphi ) + H_{eff} \sin \varphi \cos \varphi = 0 $$

where *ω* is the angular frequency, *γ* is the gyromagnetic ratio, and H_eff_ is the in-plane effective uniaxial anisotropy field. Figure 4 shows the fitted H_r_–φ curves (black lines) based on the experimental H_r_–φ curves (red dots). Figures 5 and 6 show the corresponding values of M_s_ and H_eff_ under different electric fields, respectively.

The *loop*-like dependence of M_s_ on the electric field is shown in Fig. 5. A 1.04% change of M_s_ is obtained. This result can be explained as follows: Suppose an FE layer with surface charge σ_FE_ and electric polarization **P** is brought in contact with a charge-neutral FM. The bond rearrangements occur within a few atomic layers of the interface of the FM/FE multiferroic heterostructure, which rearranges spin–polarized charge density *s* on the FM layer. The interfacial spin density can be viewed as a magnonic accumulation stabilized by the **P** of the FE layer at the coupled FE/FM interface, which modifies of the magnitude of the interfacial local magnetic moments. Here we are interested in the interfacial spin density s_||_, whose direction adiabatically follows the intrinsic magnetization **M** at an instant of time, and which is given as: *s*_||_= *ησ*_FE_*e*^-z/λ^/*λ*^16–18,20^, where η is the spin polarization of the electron density in the CoZr layer in the Stoner mean-field theory, *σ*_FE_ is the surface charge density, and λ is the effective spin diffusion length in the CoZr layer. Therefore, a major effect on the CoZr layer induced by this magnon-driven ME coupling is the change of the intrinsic magnetization, given as ΔM_||_= *ησ*_FE_*µ*_B_/*d*, with *d* being the thickness of the CoZr layer. Thus, the *loop*-like M_s_–E curve in Fig. 5 is obtained, exhibiting non-volatile behavior, which further magnifies this nonlinear interplay when the sample is measured through FMR. That is, the non-volatile change of intrinsic magnetization can be attributed to the magnon-driven ME coupling, which induces the non-volatile behavior of the H_r_–E curve in Fig. 3 and the H_eff_–E curve in Fig. 6. Moreover, the non-volatile behavior of the *loop*-like M_s_–E curve is consistent with that of P–E (polarization–electric-field) curve, which indicates that the magnetization can be modulated by reversing the electric polarization as shown in the left and right insets of Fig. 5.

**Figure 7:**
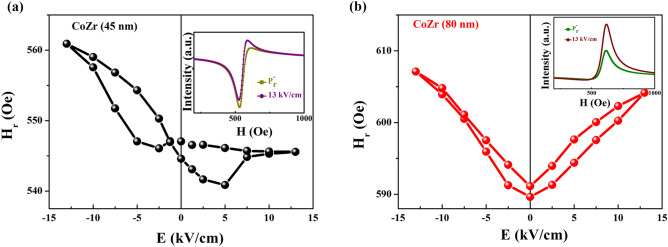
H_r_–E curves for CoZr thin film with different thicknesses. (**a**) 45-nm-thick and (**b**) 80-nm-thick CoZr thin film. The insets show the FMR integral spectra at P_r_^−^ state and under an electric field of 13.0 kV/cm.

Experimentally, several reports based on single-crystal (011) PMN-PT, such as Ni_0.46_Zn_0.54_Fe_2_O_4_/PMN-PT^7^, Ni_0.79_Fe_0.21_/PMN-PT^13^, Fe_3_O_4_/PMN-PT^22^ structure have been investigated, the magnetic properties can be mediated by piezostrain effect due to the piezoelectricity of PMN-PT. To confirm the magnon-driven ME effect rather than the piezostrain effect is a main factor in our system, CoZr thin films with thicknesses 45 nm and 80 nm have been prepared on the PMN-PT substrate in the same sputtering conditions. The result of dynamic measurement can be discussed as following. For the 45-nm-thick CoZr thin film prepared on the PMN-PT substrate, the asymmetric butterfly-like H_r_–E curve was shown in Fig. 7a, which was different from the result for 20-nm-thick CoZr thin film. The inset of Fig. 7a showed the FMR integral spectra at P_r_^−^ state and under an electric field of 13.0 kV/cm. In view of the S–E curve as shown in Fig. 1b, the piezostrain effect, derived from PMN-PT substrate, and transferred to the CoZr layer to control the resonance field demonstrating butterfly-like behavior, can be taken into consideration. The behavior of H_r_–E curve as shown in Fig. 7a can be regarded as the result of competition between piezostrain and magnon-driven interfacial ME coupling. With increasing the thickness of CoZr thin film to 80 nm, the H_r_–E curve was shown in Fig. 7b. The standard butterfly-like curve of the resonance field as a function of the electric field was obtained, which was consistent with the result of butterfly-like S–E curve of CoZr/PMN-PT structure as shown in Fig. 1b. The result indicates that the magnetic dynamics of 80-nm-thick CoZr thin film grown on PMN-PT substrate can be mainly mediated by piezostrain effect. However, for the 20-nm-thick CoZr thin film prepared on the PMN-PT substrate, the loop-like H_r_–E curve as shown in Fig. 3 exhibits non-volatile behavior, which can be attributed to magnon-driven interfacial ME coupling. The magnon-driven interfacial ME coupling can lead to the non-volatile behavior of nanometer-sized FM layer when applying electric fields. Moreover, the property of respond time of nanoseconds can be widely applied in future microwave devices in GHz frequency band.

## Conclusions

We have reported magnon-tuning non-volatile magnetic dynamics in a CoZr/PMN-PT structure. A *loop*-like curve was obtained for the resonance field versus electric field, exhibiting non-volatile behavior. This can be attributed to the magnon-driven interfacial magnetoelectric coupling. We fitted the experimental results for the resonance field versus in-plane rotating-angle under different electric fields, and obtained a *loop*-like magnetization response to the electric field. This further verifies that the magnon-driven interfacial magnetoelectric coupling has a dominant role in the CoZr/PMN-PT structure, which leads to the resulting *loop*-like curve of the effective magnetic field versus electric field. This can be applied in spintronic devices with new functions.

## Methods

### Sample preparation

The polycrystalline CoZr thin film was deposited through radio-frequency (RF) magnetron sputtering with an oblique sputtering angle of 20° on a 230-μm-thickness single-crystal (011) PMN-PT substrate. As shown in the inset of Fig. 1a, four Zr chips were regularly placed on a Co target, and the composition of the deposited magnetic layer was adjusted by controlling the number of Zr chips. The thickness of the CoZr film was 20 nm. The easy axis of the CoZr thin film during sputtering was along the [100] direction of the PMN-PT substrate. Pt layers with thicknesses of 20 nm and 100 nm were sputtered on the top and bottom surfaces to act as electrodes, respectively. Cu wires were then connected to the electrodes.

### Measurement

A biased voltage was applied a Keithley 2,410 dc power supply through the Cu wires connected to the electrodes. FMR measurements were performed using a JEOL JES-FA 300 spectrometer (power of 1 mW, X-band at 8.969 GHz). The electromagnet is used to provide a static external magnetic field. At the center of the electromagnet, a cylindrical cavity resonator is set to put sample into it. The microwave is generated by waveguide connected with the cylindrical cavity resonator, which is normal to the sample. This microwave unit can also detect the reflection. X-ray diffraction (XRD) was measured with an X’Pert X-ray powder diffractometer with Cu K_α_ radiation (1.54056 Å). Using a Sawyer Tower circuit, the hysteresis polarization–electric-field loop was taken with a computer interfaced-loop-tracer.
